# The role of kinesin KIF18A in the invasion and metastasis of hepatocellular carcinoma

**DOI:** 10.1186/s12957-018-1342-5

**Published:** 2018-02-21

**Authors:** Weiwei Luo, Minjun Liao, Yan Liao, Xinhuang Chen, Chunyan Huang, Jiyuan Fan, Weijia Liao

**Affiliations:** 1 Laboratory of Hepatobiliary and Pancreatic Surgery, Affiliated Hospital of Guilin Medical University, Guilin, 541001 Guangxi People’s Republic of China; 2 Guangxi Medical University, Nanning, 530021 Guangxi People’s Republic of China; 3 Disease Prevention and Control Center of Guilin, Guilin, 541001 Guangxi People’s Republic of China

**Keywords:** KIF18A, Hepatocellular carcinoma, Invasion; metastasis, Molecular mechanism

## Abstract

**Background:**

KIF18A is associated with a variety of tumours; however, the specific mechanism of action of KIF18A in hepatocellular carcinoma (HCC) remains unclear. In this study, in vitro and in vivo experiments were performed with the aim of exploring the potential function and molecular mechanism of kinesin KIF18A in the occurrence and development of HCC.

**Methods:**

We detected the expression of KIF18A in tumour and adjacent tissues as well as cell proliferation, cell invasion and migration in hepatoma cells after silencing KIF18A. KIF18A-silenced hepatoma cells were subcutaneously injected into nude mice to verify the tumorigenicity of KIF18A. We also detected the expression of signal pathway-related proteins in hepatoma cells after KIF18A knockdown with the aim of exploring the association between KIF18A and related signalling pathways.

**Results:**

The level of KIF18A protein was higher in liver cancer tissues than adjacent tissues. After silencing KIF18A in SMMC-7721 and HepG2 cells, cell growth was obviously inhibited; the migration and invasion abilities were significantly decreased and the in vivo tumour weight was decreased compared to the control group (0.201 ± 0.088 g vs 0.476 ± 0.126 g, *p* = 0.009). The expression of cell cycle-related protein (cyclin B1), invasion and metastasis-related proteins (MMP-7 and MMP-9) and Akt-related proteins in hepatoma cells was also decreased after knocking down KIF18A.

**Conclusions:**

KIF18A may promote proliferation, invasion and metastasis of HCC cells by promoting the cell cycle signalling pathway as well as the Akt and MMP-7/MMP-9-related signalling pathways and may serve as a new target for the diagnosis and treatment of HCC.

## Background

In recent reports, the incidence and mortality of hepatocellular carcinoma (HCC) have been shown to be on the rise [1]. There has been some progress into understanding the aetiology, epidemiology, diagnosis, treatment and survival rate of HCC, but the prognosis of HCC is still poor and its pathogenesis remains unclear. Therefore, the search for new therapeutic targets and new diagnostic indicators has become important for promoting three stage prevention and improving the survival rate of HCC.

KIF18A is a member of the kinesin superfamily. The main function of KIF18A is to regulate chromosome aggregation and suppress centromere movements, and it is involved in chromosome stability and maintains the oscillation attenuation of the mitotic spindle microtubule. Therefore, dysfunction of KIF18A can lead to chromosome instability [2–7]. Several studies have shown that KIF18A is highly expressed in most malignant tumours (renal carcinoma, breast cancer, etc.) and is associated with cell proliferation, tumour staging and the prognosis of tumour cells [8–10]. Some data suggest that HCC is associated with the following factors: cancer gene mutation, amplification, fusion; tumour suppressor gene loss of activity; genetic disequilibrium (DNA copy number variation); and abnormal regulation of DNA sequences (promoter methylation) [11, 12].

In our previous study, we performed microarray analysis, PCR and immunohistochemical detection of HCC tissues and demonstrated that KIF18A is highly expressed in HCC [13]. In this study, we explored the potential molecular mechanisms underlying the biological behavioural changes of hepatoma cells after KIF18A dysregulation to find new diagnostic targets or potential therapeutic targets for HCC. By knocking down the expression of KIF18A in hepatoma cells, we detected biological behavioural changes of HCC cells and explored the correlation between KIF18A and some signalling pathway-related proteins (Akt, Aurora A, cyclin B1, MMP-7, etc.). It was shown that KIF18A was correlated with HCC cell proliferation, invasion and migration and may be promoted by the cell cycle signalling pathway and MMP-7/MMP-9-related signalling pathway. KIF18A may serve as a new diagnostic marker and play an important role in the diagnosis and treatment of HCC.

## Methods

### Synthesis and screening of siRNA

Three pairs of small interfering RNA (siRNA) were used to knock out KIF18A and were designed and synthesised by GenePharma Con., Ltd. (Shanghai) according to the encoding sequence of human KIF18A. The sequences of the three pairs of siRNAs were as follows, siRNA-1: 5′-GUGCCACCAUAUGAAAGUATT-3′ and 5′-UACUUUCAUAUGGUGGCACTT-3′ and siRNA-2: 5′-GGUCGUUCAUGGACUUACUTT-3′ and 5′-AGUAAGUCCAUGAACGACCTT-3′. siRNA-NC (5′-GAGUUAAAGUCAAAGUGACTT-3′, 5′-GUCACUUUGACUUUAACUCTT-3′) was used as a negative control. Lipofection was performed in HCC cell lines (SMMC-7721 and HepG2) using Lipofectamine™ 3000(Invitrogen, USA). After 8 h of transfection under serum-free medium conditions, the medium was changed to DMED (New York; Grand Island, USA) containing 10% foetal bovine serum (FBS) and continuously cultured for 24 h. There were three groups included in the screening: the siRNA-KIF18A group (containing three different siRNAs), siRNA-NC group and blank control group. The total RNA of each sample was extracted 48 h after transfection. The siRNA with the best transfection efficiency was screened by detecting the expression of related genes using the real-time PCR method.

### Cell culture

We used SMMC-7721 and HepG2 cells from the Institute of chemistry and cell biology (Shanghai, China) in the preliminary studies (SMMC-7721 resistant cell lines, HepG2 for low metastasis cell line). The frozen tube containing HCC cells was removed from liquid nitrogen and then incubated in a 37 °C water bath for resuscitation. After centrifugation, the supernatant was removed and the cells were transferred into a 10-cm culture flask containing 10 ml of medium. Hepatoma cells were cultured at 37 °C in a 5% CO_2_ incubator with a culture medium containing 10% FBS and DMEM.

### Lentivirus transfection

After the previous screening, we selected siRNA-2, which had a high transfection efficiency and consisted of a recombinant vector. A recombinant lentiviral vector and packaging plasmid were co-transfected into 293T cells to obtain virus suspension. Hepatoma cells were infected by a virus suspension. Stable cell lines with stably expressed short hairpin RNA (shRNA)-KIF18A were obtained by puromycin screening, and a shRNA negative control group (shRNA-NC) was also prepared.

### Plate cloning

After silencing the expression of KIF18A, 2 ml of SMMC-7721 and HepG2 cells with a concentration of 600 cells/ml were cultured in a 6-well plate. After 14 days of culture, the cells were transferred to a 96-well plate at a concentration of 70 cells per 100 μl; then, 10 μl of cell counting kit-8 (CCK-8) was added to each well. After 2 h of culture, the absorbance at 450 nm was measured and the number of cells was calculated. The cells were washed three times with cold phosphate-buffered saline (PBS); then, they were fixed with 4% paraformaldehyde and stained with 1% crystal violet for the night. After washing, the cells were photographed with an inverted phase contrast microscope.

### Cell invasion and migration assay

Cell invasion was performed using a Matrigel Invasion Chamber (Corning, USA), and cell migration was performed using Transwell chambers (Corning, USA). Cells transfected with shRNA-KIF18A were digested with trypsin and were counted in serum-free medium. Then, 5 × 10^3^ cells per 200 μl were cultured in the upper chamber with serum-free medium, and the lower chamber was filled with DEME containing 20% foetal bovine serum. After 36 h of culture, the culture medium was removed and the cells were digested and transferred into a 96-well plate. 10 μl of CCK-8 was added to each 100-cell suspension and was gently mixed. After 2 h of culture, the absorbance at 450 nm was measured and the number of cells was calculated. After washing in cold PBS, cells were fixed with 4% paraformaldehyde and stained with 1% crystal violet overnight. The adherent cells in the inner surface of chamber were wiped with a cotton swab and photographed with an inverted phase contrast microscope, counted and analysed. In the same way, 5 × 10^3^ cells were cultured in a non-gel chamber, which was used to assess cell migration after silencing KIF18A.

### Western blotting

The total cell protein of liver cancer tissues and cancer adjacent tissues was extracted using lysis buffer (Beyotim Biotechnology) containing protease inhibitor. The tissues were oscillated 30 times per second in a tissue concussion instrument for 3 min. After 15 min of centrifugation at 4°, loading buffer was added to the supernatant and was boiled for 5 min. Protein samples were separated by 8–15% Sodium dodecyl sulfate-Polyacrylamide gel electrophoresis (SDS-PAGE) at 90 V and were transferred to polyvinylidene difluoride (PVDF) membranes (BioRad) with a 250 mA electric current.

The membranes were blocked with 5% skim milk for 1 h and were incubated with the primary antibody overnight at 4 °C. The primary antibody was the KIF18A antibody (GTX119467, Rabbit pAb, 102 kDa, dilution 1:1000, GeneTex); Akt antibody (#4685, Rabbit mAb, 60 kDa, dilution 1:1000, CST); phospho-Akt antibody (#12694, Mouse mAb, 60 KDa, dilution 1:1000, CST); MMP-7 antibody (GTX104658, Rabbit pAb, 30 kDa, dilution 1:1000, GeneTex); MMP-9 antibody (#13667, Rabbit mAb, 84 kDa, dilution 1:1000, CST); Aurora A antibody (GTX100911, Rabbit pAb, 46 kDa, dilution 1:1000, GeneTex); cyclin B1 antibody (GTX100911, Rabbit pAb, 48 kDa, dilution 1:1000, GeneTex); β-actin antibody (#12620, Rabbit mAb, 45 KDa, dilution 1:1000, CST). After washing three times with TBST (Tris buffered saline Tween-20), the membranes were incubated with a secondary antibody and then exposed on X-ray films using BeyoECL Plus (Beyotim Biotechnology).

### Nude mouse tumour model

Twelve male nude mice (5–6 weeks) were purchased from the animal experimental centre of Guilin Medical College. The mice were randomly divided into 2 groups (*n* = 12), the shRNA-KIF18A group and shRNA-NC group. HepG2 cells transfected with shRNA-KIF18A and shRNA-NC were collected in the logarithmic growth period, were cleaned in PBS and were prepared for cell suspensions. Cell suspensions (2 × 10^6^ /ml) were injected subcutaneously into the right groin region of mice. 28 days after the subcutaneous injection, mice were sacrificed by cervical dislocation. The tumour was removed, and its weight was recorded.

### Statistical analysis

All statistical analyses were performed using SPSS 18.0 (SPSS, Inc., Chicago, IL, USA). The *t* test or non-parametric test was used for the measurement data group. All data were obtained from three independent repeated experiments and are presented as the mean ± standard deviation, and *p* < 0.05 was considered to indicate statistical significance.

## Results

### Expression of KIF18A in tumour tissues and adjacent tissues

**Fig. 1:**
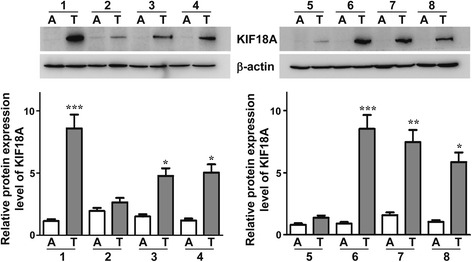
KIF18A expression in HCC tissues and adjacent tissues. The comparative method was the same sample that was repeated three times, and the *t* test was used to analyse the difference between adjacent tissues and tumour tissue. The *p* value was obtained. The expression level of KIF18A was significantly higher in tumour tissues than in adjacent tissue in 6 HCC cases, and two of them lacked a significant difference. ‘A’ indicates adjacent tissues and ‘T’ indicates tumour tissue. A vs T, *p < 0.05; **p < 0.01; ***p < 0.001

Our previous experiments demonstrated the expression of KIF18A in human liver cancer tissue and paracancerous tissue at the mRNA level, and we now show KIF18A protein expression in these tissues. There were eight cases of HCC tumour tissues and tumour adjacent tissues included in this experiment. These groups were derived from patients with HCC diagnosed by Affiliated Hospital of Guilin Medical College, and these scientific studies were approved by patients and the ethics committee. Expression of KIF18A was detected by western blot (Fig. 1), and the same sample was repeated three times. The β-actin bands remained the same in tumour and adjacent tissues, indicating that the protein content of each case was basically the same. We compared the grey values of each band, and the KIF18A band in the tumour tissues of six cases was significantly darker than that in adjacent tissues. These results indicated that the expression of KIF18A in tumour tissues was significantly higher than that in adjacent tissues.

### Relationship between KIF18A and the proliferation of hepatoma cells

**Fig. 2:**
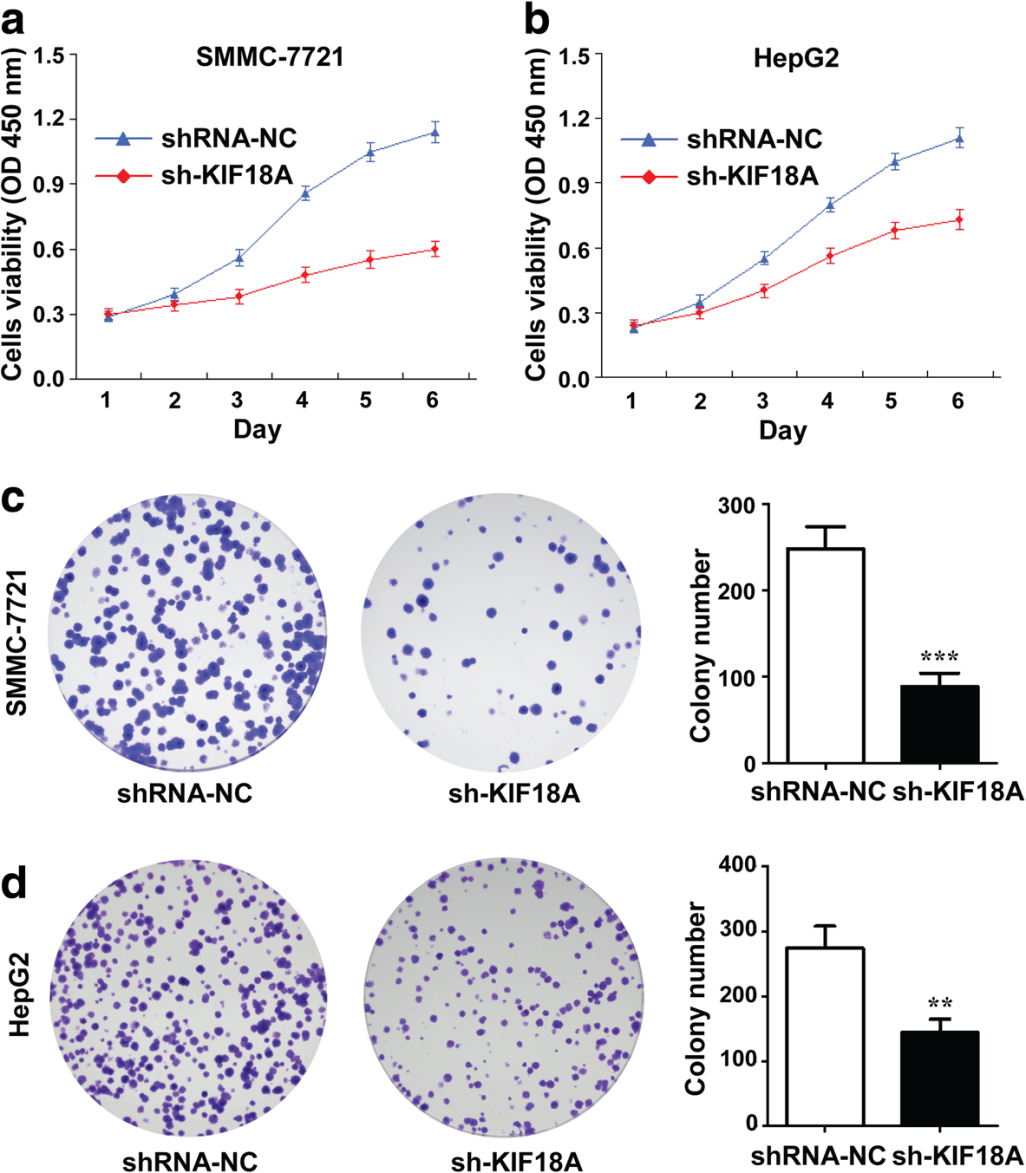
Effect of KIF18A knockdown on the proliferation of different hepatoma cells. **a**, **b**. Cell viability of HepG2 and SMMC-7721 cells transfected with shRNA-KIF18A (sh-KIF18A) and shRNA-NC. **c**, **d**. Cell proliferation status and cell clone number of HepG2 and SMMC-7721 cells transfected with shRNA-KIF18A and shRNA-NC. shRNA-KIF18A vs shRNA-NC, **p < 0.01; ***p < 0.001

We synthesised three different KIF18A siRNAs and screened the best siRNA transfection group by PCR. We selected two invasive hepatoma cell lines (SMMC-7721 and HepG2) as transfection targets for the accuracy of the experiment. Cells were cultured for 14 days after transfection, and the results of the cell activity test showed that shRNA-KIF18A transfected SMMC-7721 and HepG2 cells had lower cell viability than those in the shRNA-NC group (Fig. 2a, b) (*p* = 0.0008). The results of the flat plate clones showed that the number of minimally invasive HepG2 cells decreased to 47% and the number of highly aggressive SMMC-7721 cells decreased to 64% compared with the shRNA-NC group after transfection with shRNA-KIF18 (Fig. 2c, d) (*p* = 0.009). These results indicated that KIF18A knockdown inhibited the proliferation of HCC cells and the growth of highly invasive cancer cells. We infer that high expression of KIF18A can promote the proliferation of cancer cells and that the expression level of KIF18A is positively related to the invasiveness of different cancer cell lines.

### Relationship between KIF18A and biological activity of hepatoma cells

**Fig. 3:**
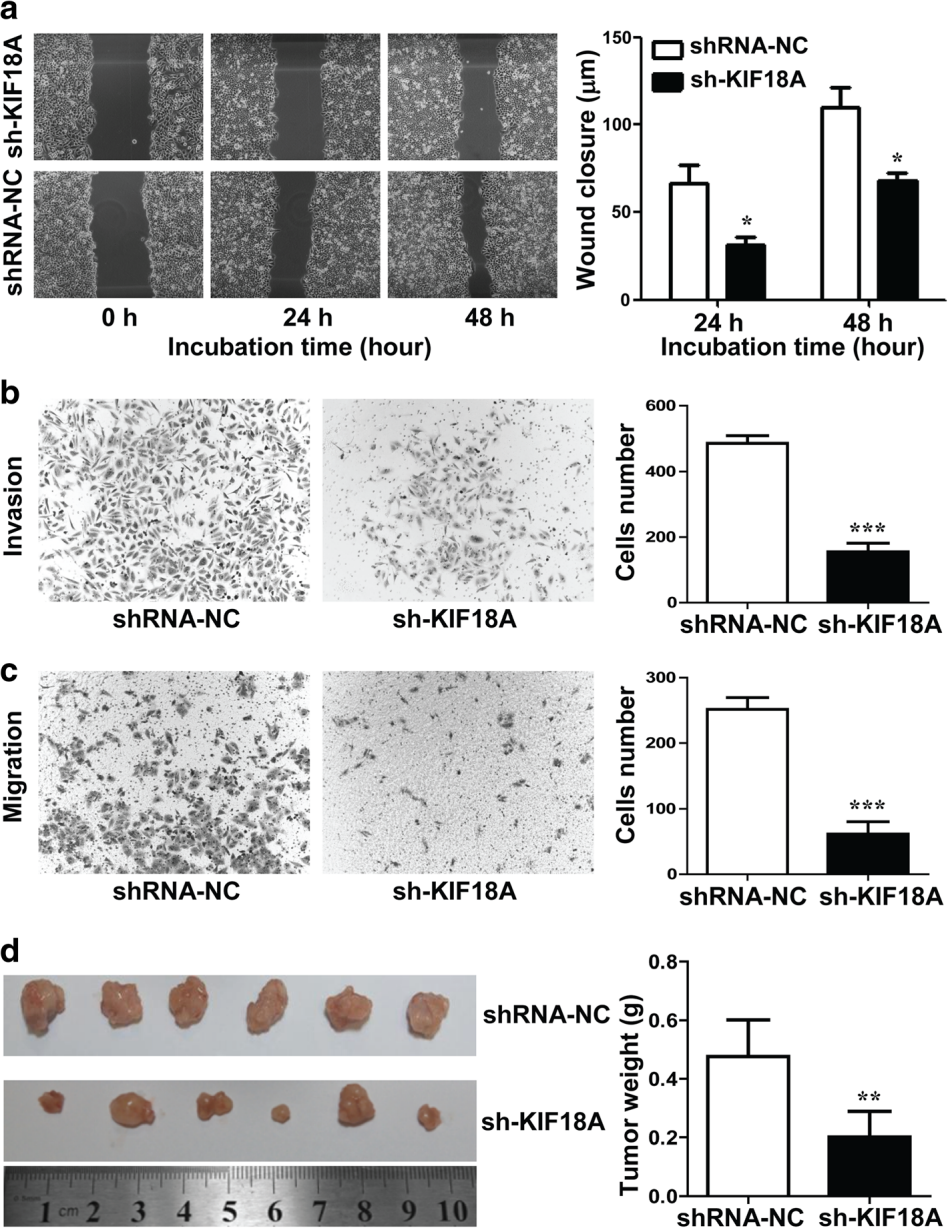
**a** Cell scratch test result of HepG2 cells transfected with sh-KIF18A and shRNA-NC after 48 h in culture. **b**, **c**. The invasion and migration of hepatoma cells after transfection with shRNA-KIF18A and shRNA-NC. **d** The tumorigenicity of HCC cells transfected with shRNA-KIF18A and shRNA-NC

To further explore the effect of KIF18A on cell proliferation, invasion and migration as well as the changes in tumorigenicity of hepatoma cells in vivo, the cell scratch test, transwell chamber assay and nude mouse tumorigenicity assay were performed. HepG2 cells transfected with shRNA-KIF18A and shRNA-NC were cultured for 24 and 48 h, and the distance between the two sides of the scratch cell was measured under an inverted phase contrast microscope. The cell scratch test results (Fig. 3a) *(p* = 0.04) showed that after 24 h of culture, the mean scratch distance of HepG2 cells transfected with shRNA-KIF18A was 30 μm and that of the shRNA-NC group was 70 μm. After 48 h of culture, the scratch distances of HepG2 cells transfected with shRNA-KIF18A and shRNA-NC were 75 and 120 μm, respectively. These results showed that the migration ability of HCC cells transfected with shRNA-KIF18A was significantly lower than that of the shRNA-NC group after 24 and 48 h in culture. These results indicated that knockdown of the KIF18A gene significantly reduced the migration ability of HCC cells.

The layer of Matrigel mimics the basal membrane of the cell. The number of cells passing through Matrigel represents the number of cells invading the stroma membrane of cancer cells in vivo, reflecting the invasive ability of cancer cells. The mean number of HepG2 cells transfected with shRNA-NC was 498, and the mean number of shRNA-KIF18A transfected HepG2 cells was 177, which decreased by 64.5% (*p* < 0.05) (Fig. 3b). In another set of experiments, the mean number of HepG2 cells transfected with shRNA-NC was 256 and the mean number for the shRNA-KIF18A group was 80, which decreased by 68.8% (*p* < 0.05) (Fig. 3c). This result indicated that the invasive ability of HepG2 cells was significantly weakened after silencing the KIF18A gene.

We cultured shRNA-KIF18A and shRNA-NC transfected HepG2 cells to the logarithmic growth phase. The cell suspensions were subcutaneously injected into the right inguinal region in mice at a concentration of 2 × 10^6^ /ml. 28 days after injection (Fig. 3d), the number of tumours in the shRNA-KIF18A group was 6/6, the number of tumours in the shRNA-NC group was 6/6, and the tumour weight of the shRNA-KIF18A group (0.201 ± 0.088 g) was significantly lighter than that of the shRNA-NC group (0.476 ± 0.126 g) (*p* = 0.009). This result showed that the tumorigenic ability of HCC cells was decreased after KIF18A knockdown.

### Association between KIF18A expression and related signalling pathways

**Fig. 4:**
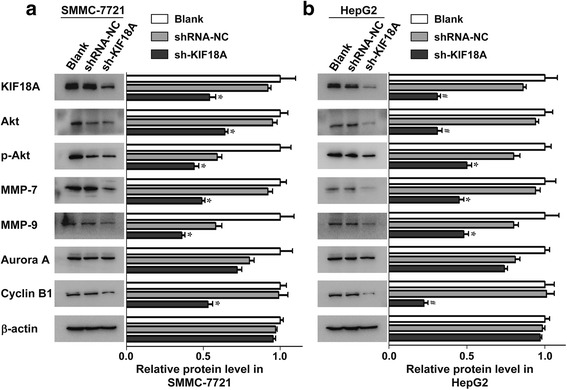
The expression of KIF18A and other signal pathway-related proteins in hepatoma cells. **a**, **b**. The expression of KIF18A, Akt, p-Akt, MMP-7, MMP-9 and cyclin B1 in sh-KIF18A transfected SMMC-7721 (**a**) and HepG2 (**b**) cells was significantly lower than the shRNA-NC group. Our comparison method is as follows: the same sample was repeated three times, and the *t* test was carried out according to the relative grey value of western blotting and the *p* value was obtained

**Fig. 5:**
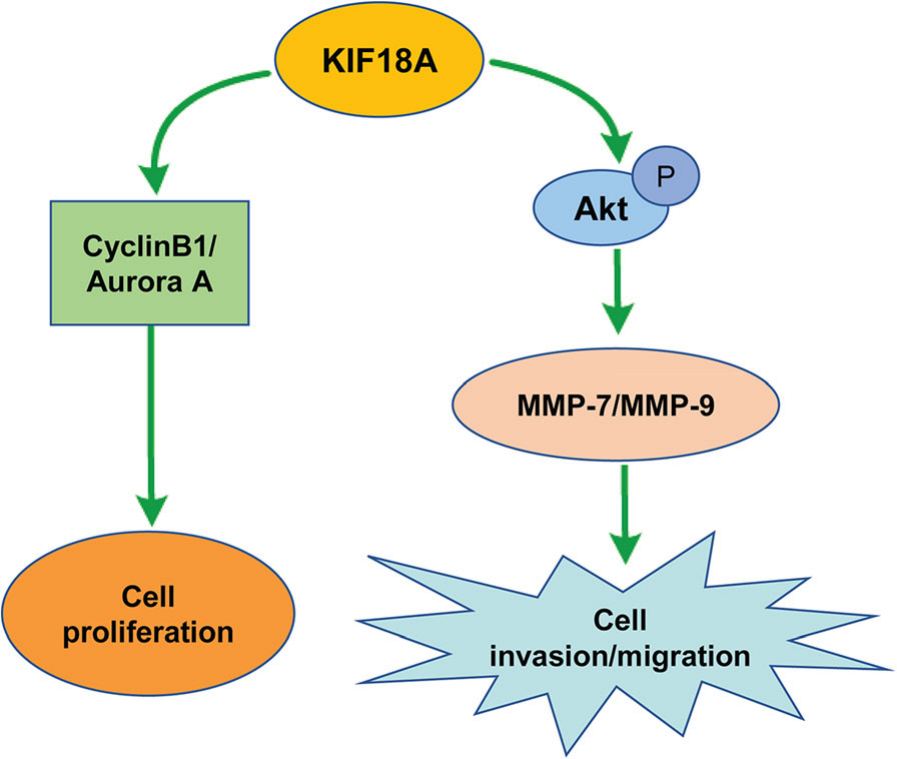
Possible signal pathway of KIF18A affecting cell proliferation, cell invasion and migration in hepatoma cells

SMMC-7721 and HepG2 cells were transfected with shRNA-KIF18A and shRNA-NC, and untreated cells served as the blank group. Western blot was used to detect the expression of signalling pathway-related proteins. The expression levels of KIF18A, Akt, p-Akt, MMP-7, MMP-9, Aurora A, cyclin B1 and β-actin were detected in each group (Fig. 4). The protein levels of KIF18A, Akt, p-Akt, MMP-7, MMP-9 and cyclin B1 were significantly lower in the shRNA-KIF18A group than in the shRNA-NC group for both SMMC-7721 and HepG2 cells. The relative mean values of the SMMC-7721 group were as follows: KIF18A (0.54), Akt (0.64), p-Akt (0.44), MMP-7 (0.49), MMP-9 (0.36), Aurora A (0.72), cyclin B1 (0.53) and β-actin (0.955). The relative mean values of the HepG2 group were as follows: KIF18A (0.31), Akt (0.31), p-Akt (0.5), MMP-7 (0.45), MMP-9 (0.48), Aurora A (0.74), cyclin B1 (0.225) and β-actin (0.97). These results showed that silencing KIF18A in hepatoma cells affected the transmission of many signalling pathways, which may promote the proliferation and metastasis of cancer cells. Based on the results above, we constructed a possible signal pathway of KIF18A that affects cell proliferation, cell invasion and migration of hepatoma cells (Fig. 5). KIF18A may promote cell invasion and migration via the Akt and MMP-7/MMP-9-related pathways and may promote cell proliferation by promoting the expression of cyclin B1.

## Discussion

HCC is a highly malignant tumour of the digestive tract and is a serious threat to human life. In recent years, its morbidity and mortality have been shown to be rising [1]. The 5-year survival rate of HCC is still not high, and surgical resection remains the primary treatment. Therefore, early diagnostic markers and possible therapeutic targets have become important components of clinical studies. Chen et al. analysed data from 295 HCC patients and found that abnormal expression of KIF is closely related to the progression and prognosis of HCC. KIF4B is considered to be an independent prognostic factor of HCC. However, the mechanisms of other genes are still unclear and require further study; also, there has been no prior analysis of KIF18A [14]. KIF18A is a super kinesin that reduces oscillations and stabilises the function of chromosome during mitosis. Studies have reported that KIF18A is highly expressed in many malignant tumours and is involved in the occurrence and development of tumours, but its mechanism is still unclear [7, 8].

In this study, we found that the expression of KIF18A in cancer tissues was higher than that of adjacent tissues. Elevated KIF18A may promote the development of HCC. To further explore the effect of KIF18A on the biological behaviour of hepatoma cells, we knocked down the KIF18A gene in two hepatoma cell lines to investigate the changes in their biological activity. The results showed that after silencing KIF18A, cell proliferation, cell invasion and migration decreased in minimally invasive HepG2 cells and highly invasive SMMC-7721 cells. For these changes, the relevant literature suggests that the KIF18A gene causes dysregulation of cell mitosis control and promotes cell division [15]; however, the mechanism remains uncertain. In one of the Przybyl studies, regulation of impaired mitosis in the early stage of the tumour can cause synsa [16]. This indicates that abnormal expression of KIF18A in liver cancer may lead to abnormal regulation of mitosis, but the specific molecular mechanism requires further research and confirmation.

To further understand the molecular mechanism of highly expressed KIF18A in HCC, we detected the expression of some proteins that are associated with cancer-related signal pathways by western blot, including cell cycle-related protein (cyclin B1), oncogene Akt, and metastasis-associated proteins (MMP-7, MMP-9). Cyclin B1 regulates mitosis in the G2/M phase of the cell cycle, and abnormalities in cyclin B1 may cause tumorigenesis [17, 18]. MMP-7 and MMP-9 are the main proteases of zinc-dependent endopeptidases and participate in extracellular matrix degradation, which is associated with the movement of tumour cells [19]. Our results showed that the levels of these proteins were significantly decreased after silencing KIF18A, and we speculated that KIF18A promotes invasion and metastasis of cancer cells by the MMP-7/MMP-9-related pathway (Figs. 4 and 5), which has accelerated cell proliferation by promoting cell cycle-related proteins. To further explore the changes in the tumour formation ability of hepatoma cells after KIF18A gene knockdown, we used HepG2 cells that were stably transfected with shRNA-KIF18A subcutaneously injected in nude mice. The rate of cell growth in the experimental group was slower than in the shRNA-NC HepG2 cell group, and the tumorigenic ability of the cells after knocking down the KIF18A gene was significantly decreased. This suggests that the expression of KIF18A can promote tumour formation in vivo.

Conventional alpha-fetoprotein (AFP) detection and imaging diagnosis have limitations in their sensitivity and specificity in primary screening. For now, there is no appropriate method to differentiate between cancer tissue and adjacent tissue or identify distant metastasis of a single cell, which has a negative influence on the extent of resection. Combining diagnostic markers with imaging, such as fluorescence or isotope labelling, KIF18A imaging may be able to accurately distinguish between cancerous tissue and adjacent tissue before surgery, allowing for advanced metastasis detection at the cellular level. Some researchers have successfully modified the expression of KIF18A in other tumours [20–24]. The development of immune blocking agents interferes with the proliferation and invasion of HCC cells from the signalling pathway, which has become the focus of future work.

## Conclusions

In conclusion, KIF18A was upregulated in HCC tissues and promoted HCC cell proliferation, invasion and migration by promoting the cell cycle signalling pathway as well as the Akt and MMP-7/MMP-9-related signalling pathways. Therefore, KIF18A may serve as a promising biomarker for the diagnosis and treatment of HCC.

## Abbreviations

CCK-8: Cell counting kit-8
FBS: Fetal bovine serum
HCC: Hepatocellular carcinoma
p: Phospho
PVDF: Polyvinylidene difluoride
SDS-PAGE: Sodium dodecyl sulfate-Polyacrylamide gel electrophoresis
shRNA: Short hairpin RNA
siRNA: Small interfering RNA
TBST: (Tris buffered saline Tween-20)
n: Number
vs: Versus
AFP: Alpha-fetoprotein
